# Bildgebung des vorderen Kreuzbands und der anterolateralen Rotationsinstabilität des Kniegelenks

**DOI:** 10.1007/s00117-024-01278-0

**Published:** 2024-03-05

**Authors:** Benjamin Fritz

**Affiliations:** 1 Abteilung für Radiologie, Universitätsklinik Balgrist, Forchstr. 340, 8008 Zürich, Schweiz; 2 Medizinische Fakultät, Universität Zürich, Zürich, Schweiz

**Keywords:** VKB-Ruptur, Magnetresonanztomographie, Knieverletzung, Knieinstabilität, Anterolaterales Ligament, ACL tear, Magnetic resonance imaging, Knee injuries, Knee instability, Anterolateral ligament

## Abstract

Das vordere Kreuzband (VKB) ist essenziell für die Stabilität des Kniegelenks. Die VKB-Ruptur stellt eine der häufigsten Sportverletzungen dar, mit einer hohen Inzidenz besonders bei Sportarten, die Drehbewegungen und abrupte Richtungswechsel erfordern. VKB-Verletzungen sind selten isoliert und oft von Meniskus- und anderen Kniebinnenverletzungen begleitet, die das Arthroserisiko erhöhen. Das Verletzungsspektrum des VKB umfasst Zerrungen, Teilrupturen und vollständige Rupturen. Die Magnetresonanztomographie (MRT) spielt eine zentrale Rolle in der Diagnostik, da sie nicht nur das VKB, sondern auch begleitende Verletzungen präzise darstellen kann. Protonendichte- und T2-gewichtete Sequenzen eignen sich besonders zur Beurteilung des VKB, welches in der Regel in allen Ebenen gut sichtbar und bewertbar ist. Neben der Darstellung der Faserunterbrechung als direktem Zeichen und zentralem diagnostischem Indikator einer VKB-Ruptur gibt es zahlreiche weitere direkte und indirekte Anzeichen einer VKB-Verletzung in der MRT. Dazu gehören abnormale Faserorientierungen, Signalerhöhungen sowie eine anteriore Subluxation der Tibia im Verhältnis zum Femur. Die häufig mit VKB-Rupturen assoziierten Knochenmarködeme sind oft hinweisend auf den zugrundeliegenden Verletzungsmechanismus. Die Therapie der VKB-Rupturen kann konservativ oder operativ sein, abhängig von verschiedenen Faktoren wie dem Aktivitätsniveau des Patienten und dem Vorhandensein von Begleitverletzungen. Die präzise und umfassende Beschreibung von VKB-Verletzungen durch die Radiologie ist entscheidend für die optimale Therapieplanung. Die anterolaterale Rotationsinstabilität (ALRI) des Kniegelenks kennzeichnet einen Zustand übermäßiger lateraler und rotatorischer Beweglichkeit der Tibia im Verhältnis zum Femur im anterolateralen Kniebereich. Diese Instabilität wird primär durch eine Ruptur des VKB verursacht, wobei dem vor etwa 10 Jahren wiederentdeckten anterolateralen Ligament (ALL) ebenfalls eine Rolle bei der Stabilisierung des Knies zugeschrieben wird. Obwohl die ALRI in erster Linie durch klinische Untersuchungen diagnostiziert wird, ist die MRT unerlässlich für das Erkennen von Verletzungen am VKB, ALL und anderen Kniebinnenstrukturen, was essenziell für die Entwicklung einer optimalen Behandlungsstrategie ist.

## Abstract (en)

The anterior cruciate ligament (ACL) is essential for the stability of the knee joint and ACL tears are one of the most common sports injuries with a high incidence, especially in sports that require rotational movements and abrupt changes in direction. Injuries of the ACL are rarely isolated and are often accompanied by meniscal and other internal knee injuries, which increase the risk of osteoarthritis. The spectrum of ACL injuries includes strains, partial tears and complete tears. Magnetic resonance imaging (MRI) plays a pivotal role in the diagnostics as it can accurately depict not only the ACL but also accompanying injuries. Proton density and T2-weighted sequences are particularly suitable for evaluating the ACL, which is usually well visible and assessable in all planes. In addition to depicting fiber disruption as a direct sign and central diagnostic indicator of an ACL tear, there are numerous other direct and indirect signs of an ACL injury in MRI. These include abnormal fiber orientations, signal increases and an anterior subluxation of the tibia relative to the femur. The bone marrow edema patterns often associated with ACL tears are indicative of the underlying injury mechanism. The treatment of ACL tears can be conservative or surgical depending on various factors, such as the patient’s activity level and the presence of accompanying injuries. The precise and comprehensive description of ACL injuries by radiology is crucial for optimal treatment planning. Anterolateral rotational instability (ALRI) of the knee joint characterizes a condition of excessive lateral and rotational mobility of the tibia in relation to the femur in the anterolateral knee region. This instability is primarily caused by a rupture of the ACL, with the anterolateral ligament (ALL) that was rediscovered about 10 years ago, also being attributed a role in stabilizing the knee. Although ALRI is primarily diagnosed through clinical examinations, MRI is indispensable for detecting injuries to the ACL, ALL, and other internal knee structures, which is essential for developing an optimal treatment strategy.

Das vordere Kreuzband (VKB) ist das am häufigsten verletzte Ligament des Kniegelenks. In den USA wird die jährliche Inzidenz der VKB-Rupturen auf über 200.000 Fälle geschätzt, während in Schweden die Inzidenz bei 78 pro 100.000 Einwohnern liegt [19, 24]. VKB-Rupturen sind typische Sportverletzungen, die vor allem bei Sportarten mit intensiven Drehbewegungen des Kniegelenks auftreten. In Deutschland sind vorrangig Athleten in Mannschaftssportarten wie Fußball, Handball oder Basketball betroffen, aber auch Skifahrer erleiden verhältnismäßig häufig VKB-Verletzungen [6]. Die meisten VKB-Verletzungen treten ohne direkte Fremdeinwirkung auf, insbesondere während abrupter Richtungswechsel und Abbremsmanöver, die zu unkontrollierten Verdrehungen und Überstreckungen des Kniegelenks führen können. Besonders Adoleszente und junge Erwachsene haben ein erhöhtes Risiko für eine VKB-Ruptur. Zudem zeigen einige Studien signifikante Geschlechtsunterschiede, wobei junge Frauen ein bis zu fünffach erhöhtes Risiko für eine VKB-Ruptur im Vergleich zu ihren männlichen Counterparts aufweisen. Die Ursachen hierfür sind vielschichtig und noch nicht vollständig geklärt, können jedoch teilweise durch Unterschiede in Anatomie, Biomechanik, Bandlaxizität sowie durch die Intensität der sportlichen Betätigung erklärt werden [23].

Die VKB-Ruptur ist nur selten eine isolierte Knieverletzung. Begleitende Meniskusrisse und andere Kniebinnenverletzungen sind häufig und tragen zu einem erhöhten Risiko für früh einsetzende posttraumatische Arthrose etwa 10 bis 15 Jahre nach der Erstverletzung bei. Klinisch äußert sich eine akute VKB-Ruptur meist mit erheblicher Knieinstabilität, Schmerzen, Schwellungen und eingeschränkter Kniefunktion. Der Stellenwert der Bildgebung liegt in der Bestätigung oder dem Ausschluss der Verdachtsdiagnose der VKB-Verletzung sowie in der Detektion von Begleitverletzungen. Dabei steht das Röntgenbild immer am Anfang der Bildgebung und ist auch laut den Richtlinien des American College of Radiology die geeignete erste bildgebende Untersuchung bei Erwachsenen und Kindern ab einem Alter von 5 Jahren nach akutem und symptomatischem Knietrauma [3]. Röntgenaufnahmen können Gelenkergüsse, Frakturen, Deformitäten und Fehlstellungen zeigen. Abhängig von der Schwere der Verletzung, den klinischen Symptomen und der Übereinstimmung der radiographischen Befunde mit den Verdachtsdiagnosen der körperlichen Untersuchung, ist eine weiterführende Schnittbildgebung angezeigt. Die MRT ist aufgrund ihres hohen Weichteilkontrasts nicht nur zur genauen Evaluation des vorderen Kreuzbands bestens geeignet, sondern erlaubt auch die Detektion von nahezu allen relevanten intra- und extraartikulären Begleitverletzungen. Manche Metaanalysen beschreiben hierbei eine Sensitivität und Spezifität der MRT für die Detektion der vollständigen VKB-Ruptur von jeweils 94–95 % [20, 25]. Die Computertomographie (CT) ist dagegen weitestgehend ungeeignet zur Detektion von VKB- und anderen Weichteilverletzungen, kann aber zur genauen Evaluation von Frakturen und präoperativen Planungen nützlich sein.

Ziel dieses Artikels ist es, einen Überblick über die wesentlichen Aspekte von VKB-Verletzungen mit einem Schwerpunkt auf der MRT-Bildgebung zu geben. Im zweiten Abschnitt wird die klinische Diagnose der anterolateralen Rotationsinstabilität des Kniegelenks dargelegt, welche maßgeblich von der Integrität des VKB abhängig ist.

## Anatomie des VKB und Biomechanik

**Figure 1:**
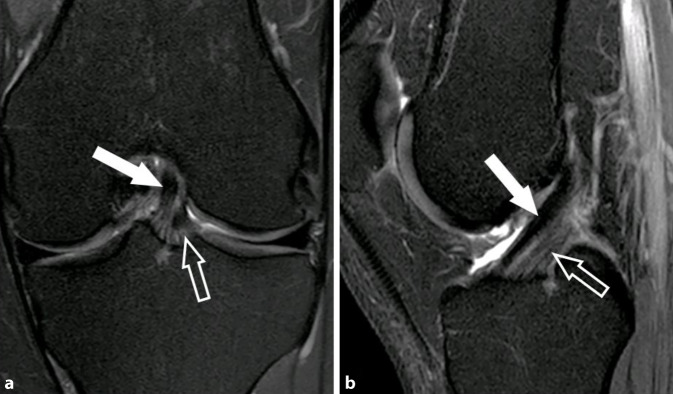

Das VKB ist ein intraartikuläres, extrasynoviales Ligament, bestehend hauptsächlich aus Typ-I-Kollagenfasern, die parallel angeordnet sind, um eine hohe Stärke und Elastizität zu gewährleisten. Das VKB verbindet das distale Femur mit der Tibia, der Ursprung befindet sich hierbei an der Innenseite des lateralen Femurkondylus und der Ansatz am anterioren Tibiaplateau im Bereich der Medianlinie. Das VKB ist aus zwei unterschiedlichen Bündeln aufgebaut, die nach ihren tibialen Ansatzstellen benannt sind: das anteromediale (AM) Bündel und das posterolaterale (PL) Bündel (Abb. 1). Der tibiale Ansatz des AM-Bündels befindet sich hierbei etwas weiter anterior und medial zum Ansatz des PL-Bündels. Der femorale Ursprung des AM-Bündels befindet sich etwas weiter kranial als der Ursprung des PL-Bündels. Das AM-Bündel ist das größere und stärkere der beiden Bündel und macht etwa 70 % der Gesamtstärke des VKB aus. Das PL-Bündel ist kleiner und trägt etwa 30 % der Stärke des VKB bei. Das PL-Bündel ist schräger orientiert als das AM-Bündel und verläuft tiefer in der interkondylären Notch. Studien haben gelegentlich ein zusätzliches, inkonstantes intermediäres Bündel beschrieben, das jedoch in der MRT-Bildgebung üblicherweise nicht zuverlässig identifiziert werden kann. Das gesamte VKB misst etwa 38 mm in der Länge und 11 mm in der Breite [11]. Das AM-Bündel ist im Mittel ca. 37 mm lang, während das PL-Bündel im Mittel ca. 21 mm lang ist. Beide Bündel sind ähnlich breit mit ca. 5 mm im mittleren Drittel [2].

Das VKB ist ein entscheidender Stabilisator im Kniegelenk. Die einzigartige Zweibündel-Anatomie spielt eine wichtige Rolle in der Biomechanik des Kniegelenks und ist entscheidend für die Kontrolle dynamischer Kniebewegungen, insbesondere bei der Bereitstellung von Rotationsstabilität und der Verhinderung einer übermäßigen anterioren Translation der Tibia gegenüber dem Femur. In Extension verlaufen die beiden VKB-Bündel parallel, wobei das PL-Bündel angespannt und das AM-Bündel relaxiert ist. Beim Übergang des Kniegelenks in Flexion gerät das AM-Bündel unter Spannung, während sich das PL-Bündel lockert. Dies wird verursacht durch eine relative Positionsänderung des femoralen Ursprungs der beiden Bündel. Gegenüber der Extension verschiebt sich in Flexion der femorale Ursprung des AM-Bündels etwas weiter nach posterior und der femorale Ursprung des PL-Bündels etwas weiter nach anterior [26]. Durch die Varianz der Spannungsverhältnisse bildet das PL-Bündel den primären Widerstand gegen die anteriore Tibiatranslation in Extension und das AM-Bündel den primären Widerstand gegen die anteriore Tibiatranslation in Flexion. Zusätzlich bietet das PL-Bündel dem Kniegelenk Rotationsstabilität während der anterioren Translation durch Anspannung sowohl bei Innen- als auch bei Außenrotation. Das VKB ist zudem reich an Mechanorezeptoren, die eine wesentliche Rolle bei der propriozeptiven Rückkopplung und neuromuskulären Kontrolle spielen. Diese Fähigkeit ermöglicht es dem Knie, auf Positions- und Belastungsänderungen zu reagieren und die Muskelaktivität entsprechend anzupassen.

## MRT-Bildgebung

Für die Bewertung der Kniebinnenstrukturen nach akuten Verletzungen wird die MRT als die bevorzugte Bildgebungsmodalität angesehen und hat die diagnostische Arthroskopie in den vergangenen 30 Jahren fast vollständig abgelöst. Die meisten Standardprotokolle ermöglichen nicht nur eine adäquate Beurteilung des VKB, sondern auch der anderen Kniebinnenstrukturen wie Menisken, Sehnen, weiteren Ligamenten und dem Gelenkknorpel. Typische klinische MRT-Protokolle bei Knieverletzungen umfassen 4 bis 5 zweidimensionale Turbo- oder Fast-Spin-Echo-Pulssequenzen in axialer, sagittaler und koronarer Orientierung mit Schichtdicken von meist um 3 mm. Nicht fettsupprimierte protonendichtegewichtete (PD) Turbo- oder Fast-Spin-Echo(TSE)-Pulssequenzen sind am vielseitigsten für die morphologische Beurteilung der Kniebinnenstrukturen, einschließlich Gelenkknorpel, Bänder, Menisken und Gelenkflüssigkeit. Fettsupprimierte flüssigkeitssensitive Pulssequenzen mit Echozeiten von 60 ms sind dagegen am besten für Kontrastbewertungen geeignet, einschließlich Gelenkflüssigkeit, Knochenmarködeme sowie ligamentäre und tendinöse Risse. T1-gewichtete Pulssequenzen können zur Verbesserung der Frakturvisualisierung verwendet werden, jedoch ist die Kombination aus PD und fettsupprimierten T2-gewichteten Pulssequenzen oft ähnlich akkurat. Eine räumliche In-Plane-Auflösung von etwa 0,5 mm ist in der Regel ausreichend um anatomische Kniestrukturen detailliert zu bewerten. Moderne Beschleunigungstechniken und künstliche Intelligenz (KI) erlauben mittlerweile schnelle Untersuchungsprotokolle des Kniegelenks von 5 min ohne relevanten Verlust der Bildqualität, was den Komfort der Patient:innen mit akuten Knieverletzungen deutlich erhöhen kann [9, 14, 16].

Das VKB ist als große Bandstruktur in Standardsequenzen meist gut beurteilbar. Es lässt sich aber aufgrund seines obliquen Verlaufs in der koronaren, sagittalen und axialen Ebene typischerweise nicht in seiner vollen Ausdehnung auf einzelnen Schichten darstellen, sondern erstreckt sich meist über mehrere Bildebenen. Zur besseren Visualisierung der Gesamtausdehnung können oblique Sequenzen mit Angulationen entlang dem schrägen VKB-Verlauf akquiriert werden. In seltenen Fällen können diese Sequenzen die Diagnostik einer VKB-Ruptur verbessern, sind aber meist für eine suffiziente VKB-Diagnostik nicht notwendig und werden aufgrund des ungünstigen Kosten-Nutzen-Verhältnisses nicht generell empfohlen. Eine Alternative hierfür stellen hochaufgelöste, isotrope 3D-Turbo- oder Fast-Spin-Echo-Pulssequenzen dar, deren Akquisitionszeiten in den letzten Jahren durch technische Innovationen auf 4 bis 5 min pro Sequenz deutlich beschleunigt werden konnten. Diese lassen sich nach Belieben multiplanar rekonstruieren und an alle Strukturen individuell anpassen, so auch an den schrägen Verlauf des VKB. Ein Protokoll bestehend aus einer isotropen PD- und einer fettsupprimierten T2-gewichteten 3D-Turbo- oder Fast-Spin-Echo-Sequenz wird in Hinblick auf die diagnostische Genauigkeit als gleichwertig gegenüber einem Standard 2D-Knieprotokoll angesehen [7, 8].

## Verletzungsmechanismen des VKB

**Figure 2:**
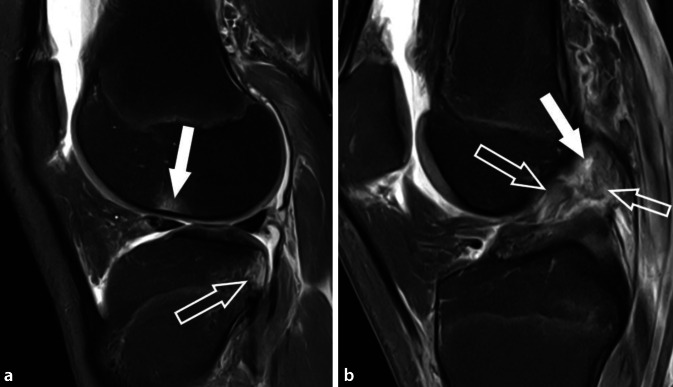

VKB-Rupturen entstehen typischerweise nicht durch exzessiven axialen Zug entlang der longitudinalen Achse des Ligaments, sondern hauptsächlich durch abnorme Scherkräfte, die bei übermäßigen Drehbewegungen auf das VKB einwirken. Dies führt oft zu Subluxationen und Kollisionen zwischen Femur und Tibia, wobei in der MRT bei über 80 % der VKB-Rupturen kontusionsbedingte Knochenmarködeme an Femur und Tibia festgestellt werden. Es wurden verschiedene Mechanismen beschrieben, die zu VKB-Verletzungen führen, welche teilweise durch ihre charakteristischen posttraumatischen Knochenmarködeme eindeutig identifiziert werden können. Der häufigste Mechanismus ist die sog. Pivot-shift-Verletzung, die in über 50 % der Fälle von VKB-Verletzungen zugrunde liegt (Abb. 2; [30]). Diese führt bei Erwachsenen, meist während sportlicher Aktivität und ohne Fremdeinwirkung, in nahezu allen Fällen zu einer hochgradigen Partialruptur oder vollständigen Ruptur der VKB. Beim Pivot-shift-Mechanismus erfährt das leicht flektierte Knie exzessiven Valgusstress in Kombination mit einer Außenrotation der Tibia. Der Fuß ist dabei fest auf dem Boden aufgesetzt. Es kommt zu einer Valgusüberlastung des Kniegelenks (oft mit Verletzung des medialen Kollateralbands) und zu einer anterioren Tibiatranslation durch Quadrizepszug, wodurch es zur VKB-Ruptur kommt. Das Kniegelenk erfährt dabei eine transiente rotatorische posterolaterale Subluxation, wodurch der laterale Femurkondylus mit dem posterolateralen Tibiaplateau kollidiert. Dabei kommt es meist zu einer flachen osteochondralen Impressionsfraktur des lateralen Femurkondylus mit charakteristischem Knochenmarködem auf Höhe des kondylopatellaren Sulkus. Diese Impression ist häufig bereits im Röntgenbild erkennbar und wird als „deep lateral femoral notch sign“ bezeichnet [21]. Im Rahmen der Knochenkollision kommt es auch im posterolateralen Tibiaplateau zu einer Knochenkontusion, welche in der MRT meist durch charakteristische Knochenmarködeme gekennzeichnet ist. Das Verletzungsspektrum reicht dabei von einfachen Knochenkontusionen mit trabekulären Mikrofrakturen bis zu dislozierten posterolateralen Tibiaplateaufrakturen mit Gelenkflächenbeteiligung.

Das Hyperextensionstrauma des Kniegelenks stellt einen weiteren Mechanismus dar, der durch ein typisches Muster von Knochenkontusionen gekennzeichnet ist. Bei der Überstreckung des Kniegelenks kommt es zur Kollision und Impaktion von Femur und Tibia, was weit anterior gelegene Knochenmarködeme an gegenüberliegenden Lokalisationen verursacht. Diese Knochenmarködeme können sowohl im lateralen als auch im medialen Kompartiment auftreten. Im Gegensatz zum Pivot-shift-Mechanismus führt der Hyperextensionsmechanismus oft nicht zu einer vollständigen Ruptur des VKB, sondern es treten hierbei relativ häufig Zerrungen und niedriggradige Partialrupturen auf.

Bei Kreuzbandverletzungen durch äußere Gewalteinwirkung, etwa durch Motorfahrzeugkollisionen oder bei Kontakt mit Gegenspielern im Sport, können die Knochenmarködeme Hinweise auf den Ort der Krafteinwirkung geben. Ein gut definierter Verletzungsmechanismus, durch welchen es zur VKB-Überlastung kommt, wie beim Pivot-shift-Mechanismus, ist bei diesen oft komplexen Hochenergietraumata jedoch häufig nicht nachvollziehbar.

## MRT-Darstellung der VKB-Rupturen

Das normale VKB ist eine in allen Sequenzen hypointense Struktur mit dicht gepackten, parallel angeordneten Faserzügen. Das PL-Bündel erscheint hier häufig etwas lockerer angeordnet als das AM-Bündel mit teils feiner linearer Streifung und gering angehobenem T1- und T2-Signal (Abb. 1). Der Verlauf des VKB ist parallel oder in gering spitzem Winkel zur Blumensaat-Linie, welche das Dach der interkondylären Notch beschreibt. Im Fall einer vollständigen VKB-Ruptur lassen sich die unterbrochenen Faserzüge meist in der sagittalen, axialen und koronalen Ebene gut abgrenzen. Hierbei verliert das VKB seine nahezu perfekt angeordnete, parallel gestreifte Architektur. Die rupturierten Fasern zeigen typischerweise eine Abweichung ihrer Orientierung entlang der Blumensaat-Linie mit gewellter Konfiguration, was als sicherer Hinweis auf eine VKB-Ruptur gewertet werden kann (Abb. 2; Tab. 1). Durch assoziierte Ödeme und Hämatome kommt es zu einer Signalanhebungen des VKB in flüssigkeitssensitiven Sequenzen sowie häufig zu einer Verdickung des Ligaments. Hier bestehen in der MRT bildmorphologische Überlappungen mit der mukoiden Degeneration des VKB, was ebenfalls mit einer PD- und T2-hyperintensen Signalalteration und Ligamentverdickung einhergeht. Die mukoide Degeneration ist jedoch typischerweise mit kleinen Ganglien an den VKB-Enden oder innerhalb der Sehnensubstanz assoziiert sowie einer intakten und parallelen Faserarchitektur. Bei hochgradigen Partialrupturen und vollständigen VKB-Rupturen kann es zu einer Dislokation des tibialen Sehnenstumpfes nach anterior mit Interposition zwischen anteriorer Eminentia intercondylaris und femoraler Notch kommen. Dadurch kann eine Extensionshemmung des Kniegelenks hervorrufen werden.

**Table 1:** 

Normale Darstellung des VKB	Direkte Zeichen der VKB-Verletzung	Indirekte Zeichen der VKB-Verletzung
Hypointense Struktur in allen Sequenzen	Unterbrechung von VKB-Fasern	Knochenmarködeme mit Kontusionsmuster
Parallele Faserarchitektur mit feiner Streifung	Achsabweichung der Faserorientierungen von der Blumensaat-Linie	PD und T2-hyperintese Signalveränderung der VKB-Substanz
VKB-Verlauf nahezu parallel zur Blumensaat-Linie	Umgeschlagene Ligamentanteile, (insbesondere distaler Sehnenanteile nach anterior)	Verdickung der VKB-Substanz
		Anteriore Subluxation der Tibia gegenüber Femur
		Posteriore Dislokation des Außenmeniskushinterhorns
		Deformierung des hinteren Kreuzbands mit Steilstellung
		Assoziierte Kniebinnenverletzungen (Meniskus, Kollateralbänder, ALL-Verletzung und Segond-Fraktur)
		Meniskokapsuläre Separation oder „ramp lesion“

Indirekte Zeichen einer VKB-Ruptur können assoziierte Verletzungen von Kniebinnenstrukturen oder femorotibialen Alignmentstörungen darstellen (Tab. 1). Häufige Begleitverletzungen einer VKB-Ruptur sind insbesondere der Riss des Innenmeniskus und des medialen Kollateralbands, was auch als „O’Donoghue unhappy triad“ bezeichnet wird. Der Riss des Innenmeniskus ist hierbei meist vertikal orientiert und im Hinterhorn gelegen. Jedoch findet sich bei einer VKB-Ruptur mit ähnlicher Häufigkeit auch ein Riss des Außenmeniskus, ebenfalls meist vertikal orientiert und im Hinterhorn lokalisiert.

In den letzten Jahren hat die Ruptur der Anheftung des Innenmeniskushinterhorns an die posteromediale Gelenkkapsel zunehmend Beachtung gefunden. Diese Verletzung, die uneinheitlich als meniskokapsuläre Separation oder „ramp lesion“ bezeichnet wird, scheint stark mit einer VKB-Ruptur assoziiert zu sein. Die Sensitivität und Spezifität der MRT für die Detektion der meniskokapsulären Separation ist wahrscheinlich niedrig. Eine vermehrte Flüssigkeitskollektion und Distanz zwischen Innenmeniskushinterhorn und posteromedialer Gelenkkapsel mit fehlender Abgrenzbarkeit der generell schlecht einsehbaren feinen meniskokapsulären oder meniskotibialen Zügel können in der MRT hinweisend auf eine meniskokapsuläre Separation sein. Des Weiteren bestehen hochgradige Assoziationen zwischen der Segond-Fraktur und der Ruptur des anterolateralen Ligaments (ALL) mit VKB-Verletzungen, worauf im letzten Teil dieses Artikels näher eingegangen wird.

**Figure 3:**
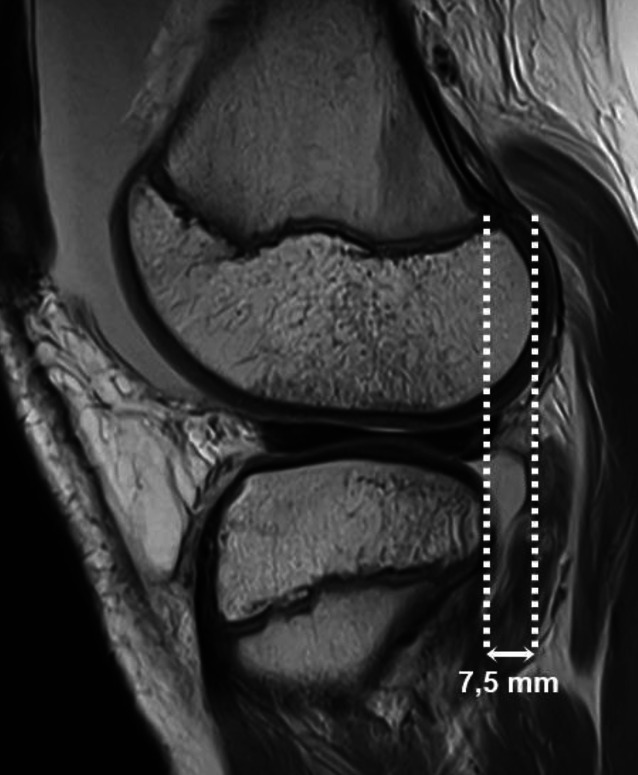

Im Rahmen der VKB-Ruptur kann es zu Alignmentstörungen mit anteriorer Subluxation der Tibia gegenüber dem Femur kommen. Dies kann vermutet werden, wenn in der zentralen sagittalen Schicht des lateralen Kompartiments die posteriore Kante der Tibia um mehr als 5 mm weiter ventral verläuft gegenüber der posterioren Kante der Femurkondyle (Abb. 3). Hierdurch kann es zu einer posterioren Dislokation des Außenmeniskushinterhorns gegenüber dem lateralen Tibiaplateau kommen. Im Rahmen der vermehrten anterioren Translation der Tibia gegenüber dem Femur kommt es zudem meist zu einer Verformung des hinteren Kreuzbands mit relativer Steilstellung und Verlust der charakteristischen hakenförmigen Konfiguration. Als weitere indirekte Hinweise auf eine VKB-Ruptur werden zudem die zuvor beschriebenen ossären Kontusionsödeme gezählt.

## Klassifikation der VKB-Ruptur

Die Kategorisierung der VKB-Verletzung in den Grad der Faserruptur ist von hoher klinischer Relevanz, da sie direkten Einfluss auf die Therapie haben kann. Dabei reicht das Spektrum der VKB-Verletzungen von Zerrungen, über Partialrupturen bis zu vollständigen Rupturen.

Der im deutschsprachigen Raum gebräuchliche Begriff „Zerrung“ steht histopathologisch für interstitielle Mikrorisse in den Ligamentfasern, die makroskopisch nicht differenzierbar sind. In der MRT äußern sich Zerrungen durch PD- und T2-Signalerhöhungen der VKB-Fasern, die mit einer möglichen Verdickung des Ligaments einhergehen können. Allerdings gibt es umfangreiche bildmorphologische Überschneidungen mit normaler Variabilität der VKB-Signale oder mit leichten mukoiden Degenerationen, die eine zuverlässige Erkennung der VKB-Zerrung in der klinischen Praxis oft erschweren. Da eine Zerrung des VKB jedoch nicht zu Knieinstabilität führt, besitzt eine übersehene Diagnose in der klinischen Praxis oft keine gravierende klinische Bedeutung.

Partialrupturen des VKB können ein Spektrum von niedrig- bis hochgradige Schädigungen umfassen. Die prozentuale Bestimmung des Ausmaßes der durchtrennten Ligamentfasern kann wertvolle Informationen für den Entscheid zwischen konservativer und operativer Behandlung liefern. Oft sind beide Bündel des VKB von einer Partialruptur betroffen, obwohl auch einzelne Bündel isoliert geschädigt sein können. Die Genauigkeit der MRT in der Erkennung von Partialrupturen des VKB ist geringer als bei der Erkennung kompletter Rupturen. Obwohl nur begrenzte Daten verfügbar sind, ergab eine Studie, dass die Sensitivität zwischen 40 und 75 % sowie die Spezifität zwischen 62 und 89 % beträgt [27].

Die vollständige Ruptur des VKB beschreibt eine komplette Durchtrennung aller Sehnenfasern. Tatsächlich zeigt sich bei der Arthroskopie häufig, dass bei MR-tomographisch vermuteten vollständigen Rupturen des VKB oft noch filiforme Faserzügel in Kontinuität erhalten sind. Daher entsprechen diese Fälle formal hochgradigen Partialrupturen. Dies beeinflusst jedoch in der Regel nicht das Management der Patient:innen, da solche Rupturen bei der Abwägung zwischen konservativer und operativer Therapie meist keinen entscheidenden Einfluss haben. Im Fall einer Operation werden hochgradige Partialrupturen, ebenso wie vollständige VKB-Rupturen, meist mittels Ersatzplastik behandelt. Mehrere Metaanalysen konnten eine hohe diagnostische Genauigkeit der MRT für die Detektion der vollständigen VKB-Rupturen nachweisen mit Sensitivtäten und Spezifitäten von jeweils ca. 94–95 % [20, 25]. Einige Studien, die von spezialisierten muskuloskeletalen Radiolog:innen durchgeführt wurden, konnten noch höhere Ergebnisse erzielen, mit nahezu perfekten Sensitivitäten und Spezifitäten von jeweils 98–100 % [4, 10]. Dadurch gehört die vollständige VKB-Ruptur zu den Verletzungen, die in der muskuloskeletalen MRT-Bildgebung am genauesten detektierbar sind.

**Figure 4:**
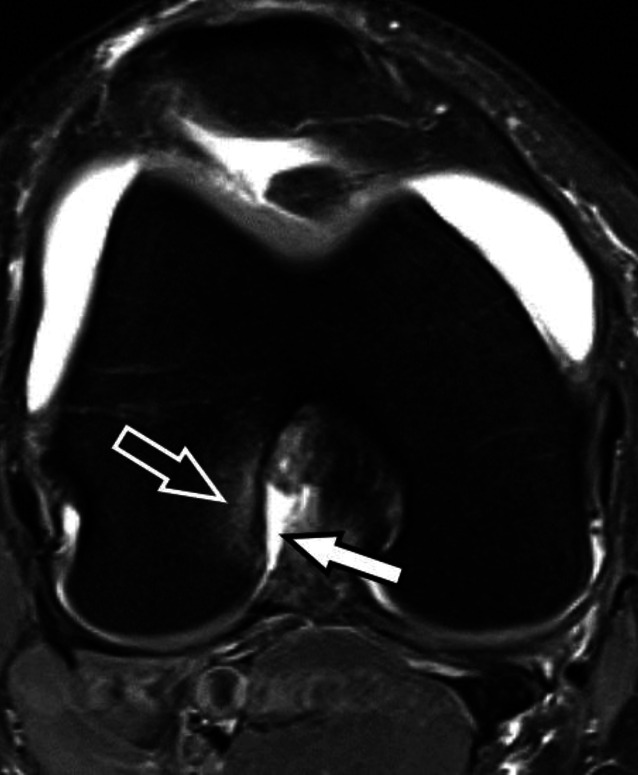

Aufgrund eines Wiederauflebens der primären VKB-Reparatur unter Erhaltung des nativen Ligaments, hat die genaue Beschreibung und Visualisierung der Höhe der Faserruptur in manchen Institutionen wieder an Relevanz gewonnen. Es konnte gezeigt werden, dass sich über die Hälfte der VKB-Rupturen in den mittleren 50 % der Ligamentausdehnung befinden. Rupturen in den distalen 25 % des VKB sind dagegen äußerst selten mit ca. 5 %. VKB-Rupturen in den proximalen 25 % finden sich dagegen bei 43 % der Patient:innen. Von besonderem Interesse sind hierbei die Rupturen der proximalen 10 % der Ligamentsubstanz, welche Avulsionen des femoralen VKB-Ursprung entsprechen. Diese scheinen sich für eine primäre VKB-Reparatur zu eignen und entsprechen etwa 16 % aller VKB-Rupturen (Abb. 4; [28]).

**Figure 5:**
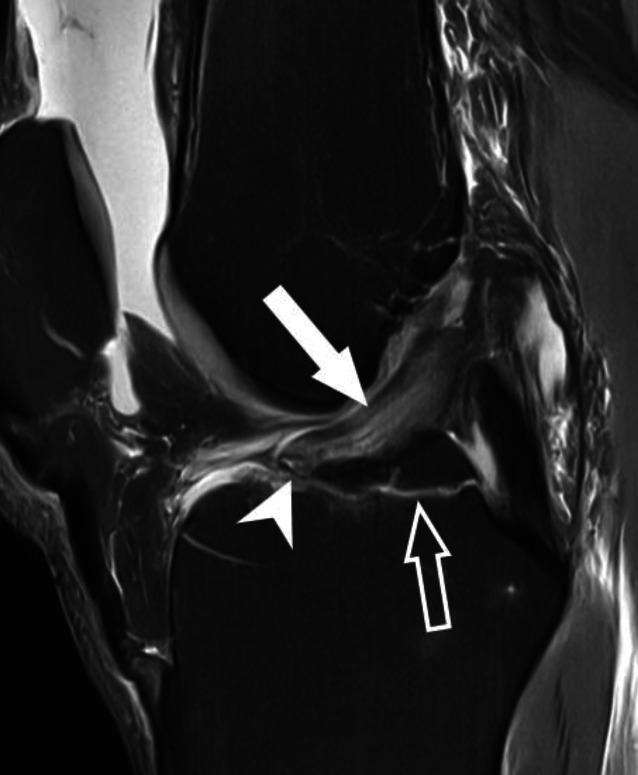

Zum Spektrum der VKB-Rupturen gehören auch die knöchernen Avulsionsfrakturen der Eminentia intercondylaris. Hierbei kommt es aufgrund des VKB-Zugs zum Ausriss von tibialen Knochenfragmenten variabler Größe, welche meist eine geringe kraniale Dislokation von wenigen Millimetern aufweisen. Das VKB bleibt hierbei meist intakt oder zeigt lediglich eine Zerrung oder niedriggradige Partialruptur. Hauptsächlich finden sich die ossären Avulsionsfrakturen der Eminentia intercondylaris bei Kindern und Adoleszenten, können aber selten auch bei Erwachsenen mit abgeschlossener Skelettreifung vorkommen (Abb. 5).

## Therapie der VKB-Rupturen

Die Therapie der VKB-Rupturen umfasst konservative und operative Methoden, in beiden Fällen mit dem Ziel der Wiedererlangung der Kniestabilität und der Reduktion des Arthroserisikos. Bei der Entscheidungsfindung sind u. a. das Aktivitätslevel, das Alter und die Begleitverletzungen von hoher Relevanz [15]. Patient:innen mit isolierten VKB-Rupturen und geringer sportlicher oder körperlicher Beanspruchung eignen sich für die konservative Therapie. Ein hohes körperliches Aktivitätslevel und/oder relevante Begleitverletzungen, wie insbesondere instabile oder rekonstruierbare Meniskusrisse, werden häufig der operativen Therapie zugeführt. Die Aufgabe der Radiologie besteht darin, mittels präziser und umfassender Beschreibung der VKB-Ruptur und der Begleitverletzungen die Voraussetzung für eine optimale Therapie zu schaffen.

## Anterolaterale Rotationsinstabilität des Kniegelenks

Die anterolaterale Rotationsinstabilität (ALRI) des Kniegelenks beschreibt einen pathologischen Zustand, der sich durch eine beeinträchtigte Stabilität zwischen Femur und Tibia, vor allem in anterolateraler Richtung, auszeichnet. Dieses abnorme Bewegungsmuster führt klinisch zu episodischer Knieinstabilität, besonders bei Aktivitäten mit Rotationsbewegungen, und ist von Symptomen wie Schmerzen, Schwellungen und dem subjektiven Empfinden eines instabilen Knies begleitet. Typischerweise zeigt sich eine abnormale und komplexe dreidimensionale Beweglichkeit, die Translation und Rotation entlang einer spiralförmigen Achse umfasst [12]. Bei der körperlichen Untersuchung zeigt sich am häufigsten eine vermehrte Innenrotation der Tibia im Vergleich zum Femur. Aus radiologischer Sicht sollte beachtet werden, dass es sich hier nicht um eine Diagnose handelt, die primär auf bildgebenden Verfahren basiert, sondern vielmehr um eine, die aus der Kombination von Anamnese, körperlicher Untersuchung und Weichteilverletzungen resultiert, die wiederum typischerweise in der MRT-Bildgebung erkannt werden.

Die ALRI wird durch eine Beeinträchtigung der strukturellen Integrität des stützenden Apparats des Knies, insbesondere der Bänder, verursacht, wobei das VKB am häufigsten betroffen ist. So kann eine ALRI des Kniegelenks nicht nur bei akuten Knieverletzungen mit VKB-Ruptur vorkommen, sondern auch bei Patienten nach insuffizienter VKB-Rekonstruktion [18]. Aber auch Verletzungen des lateralen Kollateralbands, des anterolateralen Ligaments (ALL), des Tractus iliotibialis und der Gelenkkapsel können zu einer ALRI des Kniegelenks beitragen. Diese Strukturen werden uneinheitlich von manchen Autoren als anterolateraler Komplex zusammengefasst. Inwiefern diese Strukturen des anterolateralen Komplexes zur ALRI des Kniegelenks beitragen, wird kontrovers diskutiert und hängt mutmaßlich vom Flexionsgrad des Kniegelenks ab. Studienergebnisse suggerieren jedoch, dass eine ALRI des Kniegelenks mit pathologisch erhöhter Innenrotation der Tibia gegenüber dem Femur nur im Rahmen einer Insuffizienz des VKB möglich ist, da die Durchtrennung der anterolateralen Strukturen des Kniegelenks bei intaktem VKB keine signifikante tibiale Innenrotation gegenüber dem Femur hervorruft [17].

**Figure 6:**
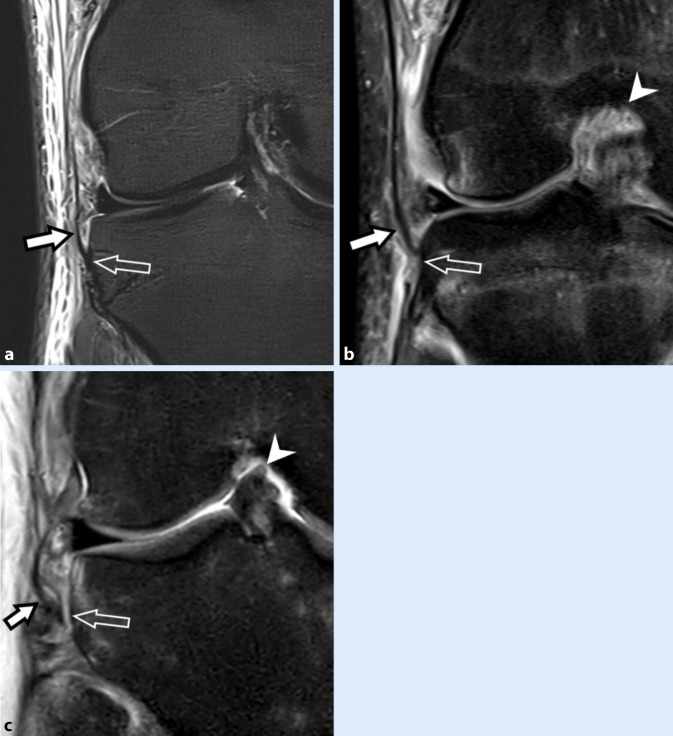

Das ALL hat seit der Beschreibung von Claes et al. im Jahr 2013 starke Beachtung gefunden [1]. Die Autorengruppe beschrieb hierbei an anatomischen Präparaten ein rundliches Ligament, welches vom lateralen Epikondylus des Femurs (anterior des Ursprungs des lateralen Kollateralbands) zum anterolateralen Tibiakopf verläuft und posterior des distalen Tractus iliotibialis und Gerdy-Tuberkulums inseriert. Es wird angenommen, dass das ALL dem anatomischen Korrelat entspricht, welches die ossäre Avulsion des kleinen kortikalen Fragments der Segond-Fraktur am anterolateralen Tibiakopf verursacht. Auch wenn die Autorengruppe in ihrer Studie das ALL als rundliches Ligament beschreibt, morphologisch ähnlich dem lateralen Kollateralband, so lässt sich dies radiologisch in der MRT typischerweise nicht nachvollziehen. Das ALL lässt sich eher als eine flache Struktur identifizieren, die signalarm in PD- und T2-gewichteten Aufnahmen erscheint und in die anterolaterale Gelenkkapsel integriert sowie mit ihr kontinuierlich verbunden ist. Deshalb ist fraglich, ob es sich hierbei tatsächlich um ein eigentliches Ligament oder eine kapsuloligamentäre Verstärkung der anterolateralen Kniegelenkkapsel handelt [22]. Im Fall der akuten Verletzung demarkiert sich das ALL aufgrund einer ödematösen Verdickung und umgebender Weichteilödeme oft deutlicher im Bereich der tibialen Insertion, wo ALL-Verletzungen typischerweise zu finden sind [5]. Das Verletzungsspektrum umfasst auch hier wie bei anderen kapsuloligamentären Verletzungen Zerrungen, Partialrupturen und vollständige Rupturen (Abb. 6).

Analog zu Segond-Frakturen, welche nahezu immer mit einer VKB-Ruptur vergesellschaftet sind, findet sich auch eine hochgradige Assoziation zwischen ALL-Verletzungen und VKB-Rupturen, primär im Rahmen von Knieverletzungen mit einem Pivot-shift-Mechanismus [29]. Auch wenn der Beitrag eines intakten ALL zur ALRI des Kniegelenks nicht vollständig geklärt ist und möglicherweise erst in Flexion an Relevanz gewinnt, so findet das ALL doch zunehmend operative Beachtung mit chirurgischer Rekonstruktion und dem Ziel, eine physiologische Kinematik und Stabilität des Kniegelenks wiederherzustellen [13].

Die radiologische Bildgebung umfasst bei Patienten mit komplexen Knietraumata und dem klinischen Bild einer ALRI immer ein Röntgenbild in 2 Ebenen zum Ausschluss von ossären Verletzungen und Degenerationen sowie meist eine MRT zur Evaluation der Weichteilverletzungen. Mittels axialen, koronalen und sagittalen Standardebenen und PD- und T2-gewichteten Sequenzen mit und ohne Fettsuppression lassen sich alle relevanten Strukturen mit besonderer Beachtung des VKB, des lateralen Kollateralbands und des ALL suffizient einschätzen und den Verdacht einer ALRI des Kniegelenks weiter erhärten oder entkräften. Ultraschall kann unter Umständen einen diagnostischen Beitrag leisten, insbesondere zur Evaluation des gut darstellbaren lateralen Kollateralbands, des äußerst selten verletzten distalen Tractus iliotibialis oder unter Umständen auch des ALL. Aufgrund der Limitationen in der Diagnostik der Kreuzbänder, der Menisken und des Gelenkknorpels sollte der Ultraschall jedoch bei vermuteter ALRI oder nach komplexem Knietrauma nicht primär in Erwägung gezogen werden.

## Fazit für die Praxis

- Das vordere Kreuzband (VKB) ist eines der am häufigsten verletzten Ligamente im Knie, besonders bei Sportarten mit intensiven Drehbewegungen und abrupten Richtungswechseln.
- VKB-Rupturen treten selten isoliert auf und sind oft von Meniskus- und anderen Kniebinnenverletzungen begleitet.
- Die Magnetresonanztomographie (MRT) ist entscheidend für die Diagnose von VKB-Verletzungen und ermöglicht die präzise Darstellung des VKB sowie begleitender Verletzungen.
- Verletzungen des VKB reichen von Zerrungen und Partialrupturen bis zu vollständigen Rupturen.
- Die MRT deckt direkte und indirekte Zeichen der VKB-Verletzung auf, einschließlich Faserunterbrechungen, Knochenmarködeme und Veränderungen in der Faserorientierung.
- Die anterolaterale Rotationsinstabilität (ALRI) des Kniegelenks beschreibt eine beeinträchtigte Stabilität des Kniegelenks, hauptsächlich verursacht durch VKB-Rupturen.
- Dem anterolateralen Ligament wird eine Bedeutung bei der Stabilisierung des Knies und der ALRI zugemessen, und es ist eng mit VKB-Rupturen assoziiert.

## References

[1] Claes S, Vereecke E, Maes M (2013). Anatomy of the anterolateral ligament of the knee. J Anat.

[2] Cohen SB, Vanbeek C, Starman JS et al (2009) MRI measurement of the 2 bundles of the normal anterior cruciate ligament. Orthopedics 32:10.3928/01477447-20090728-35 19750997

[3] Taljanovic MS, Chang EY, Expert Panel on Musculoskeletal I (2020). ACR Appropriateness Criteria(R) Acute Trauma to the Knee. J Am Coll Radiol.

[4] Fritz B, Fritz J (2022). Artificial intelligence for MRI diagnosis of joints: a scoping review of the current state-of-the-art of deep learning-based approaches. Skelet Radiol.

[5] Fritz B, Fritz J (2023). MR imaging of acute knee injuries: systematic evaluation and reporting. Radiol Clin North Am.

[6] Fritz B, Parkar AP, Cerezal L (2020). Sports imaging of team handball injuries. Semin Musculoskelet Radiol.

[7] Fritz J, Ahlawat S, Fritz B (2019). 10-min 3D turbo spin echo MRI of the knee in children: arthroscopy-validated accuracy for the diagnosis of internal derangement. J Magn Reson Imaging.

[8] Fritz J, Fritz B, Thawait GG (2016). Three-dimensional CAIPIRINHA SPACE TSE for 5-minute high-resolution MRI of the knee. Invest Radiol.

[9] Fritz J, Fritz B, Zhang J (2017). Simultaneous multislice accelerated turbo spin echo magnetic resonance imaging: comparison and combination with in-plane parallel imaging acceleration for high-resolution magnetic resonance imaging of the knee. Invest Radiol.

[10] Germann C, Marbach G, Civardi F (2020). Deep convolutional neural network-based diagnosis of anterior cruciate ligament tears: performance comparison of homogenous versus heterogeneous knee MRI cohorts with different pulse sequence protocols and 1.5-T and 3-T magnetic field strengths. Invest Radiol.

[11] Girgis FG, Marshall JL, Monajem A (1975) The cruciate ligaments of the knee joint. Anatomical, functional and experimental analysis. Clin Orthop Relat Res: 216–231 10.1097/00003086-197501000-00033 1126079

[12] Hughes JD, Rauer T, Gibbs CM (2019). Diagnosis and treatment of rotatory knee instability. J Exp Orthop.

[13] Kelly SR, Cutter BM, Huish EG (2021). Biomechanical effects of combined anterior cruciate ligament reconstruction and anterolateral ligament reconstruction: a systematic review and meta-analysis. Orthop J Sports Med.

[14] Kijowski R, Fritz J (2023). Emerging technology in musculoskeletal MRI and CT. Radiology.

[15] Kohn L, Rembeck E, Rauch A (2020). Anterior cruciate ligament injury in adults : diagnostics and treatment. Orthopade.

[16] Lin DJ, Walter SS, Fritz J (2023). Artificial intelligence-driven ultra-fast superresolution MRI: 10-fold accelerated musculoskeletal turbo spin echo MRI within reach. Invest Radiol.

[17] Lipke JM, Janecki CJ, Nelson CL (1981). The role of incompetence of the anterior cruciate and lateral ligaments in anterolateral and anteromedial instability. A biomechanical study of cadaver knees. J Bone Joint Surg Am.

[18] Musahl V, Herbst E, Burnham JM (2018). The anterolateral complex and anterolateral ligament of the knee. J Am Acad Orthop Surg.

[19] Nordenvall R, Bahmanyar S, Adami J (2012). A population-based nationwide study of cruciate ligament injury in Sweden, 2001–2009: incidence, treatment, and sex differences. Am J Sports Med.

[20] Oei EH, Nikken JJ, Verstijnen AC (2003). MR imaging of the menisci and cruciate ligaments: a systematic review. Radiology.

[21] Pao DG (2001). The lateral femoral notch sign. Radiology.

[22] Porrino J, Maloney E, Richardson M (2015). The anterolateral ligament of the knee: MRI appearance, association with the Segond fracture, and historical perspective. AJR Am J Roentgenol.

[23] Renstrom P, Ljungqvist A, Arendt E (2008). Non-contact ACL injuries in female athletes: an International olympic committee current concepts statement. Br J Sports Med.

[24] Salzler M, Nwachukwu BU, Rosas S (2015). State-of-the-art anterior cruciate ligament tears: a primer for primary care physicians. Phys Sportsmed.

[25] Smith TO, Lewis M, Song F (2012). The diagnostic accuracy of anterior cruciate ligament rupture using magnetic resonance imaging: a meta-analysis. Eur J Orthop Surg Traumatol.

[26] Torabi M, Fu F, Luo J (2013). Clinical relevance and imaging features of isolated single bundle anterior cruciate tear and single bundle augmentation. Clin Imaging.

[27] Umans H, Wimpfheimer O, Haramati N (1995). Diagnosis of partial tears of the anterior cruciate ligament of the knee: value of MR imaging. AJR Am J Roentgenol.

[28] Van Der List JP, Mintz DN, Difelice GS (2017). The location of anterior cruciate ligament tears: a prevalence study using magnetic resonance imaging. Orthop J Sports Med.

[29] Van Dyck P, Clockaerts S, Vanhoenacker FM (2016). Anterolateral ligament abnormalities in patients with acute anterior cruciate ligament rupture are associated with lateral meniscal and osseous injuries. Eur Radiol.

[30] Yoon KH, Yoo JH, Kim KI (2011). Bone contusion and associated meniscal and medial collateral ligament injury in patients with anterior cruciate ligament rupture. J Bone Joint Surg Am.

